# An Atypical Case of Monomicrobial *Clostridioides difficile* Septicemia With No Gastrointestinal Manifestations

**DOI:** 10.3389/fcimb.2022.853252

**Published:** 2022-03-31

**Authors:** Liqian Wang, Danyang Li, Zixi Chen, Liuqing He, Xianjun Wang, Liang Tao

**Affiliations:** ^1^ Affiliated Hangzhou First People’s Hospital, Zhejiang University School of Medicine, Hangzhou, China; ^2^ Key Laboratory of Structural Biology of Zhejiang Province, School of Life Sciences, Westlake University, Hangzhou, China; ^3^ Center for Infectious Disease Research, Westlake Laboratory of Life Sciences and Biomedicine, Hangzhou, China; ^4^ Institute of Basic Medical Sciences, Westlake Institute for Advanced Study, Hangzhou, China

**Keywords:** *C. difficile*, septicemia, CDI, oral trauma, monomicrobial infection

## Abstract

An uncommon case of monomicrobial *Clostridioides difficile* septicemia in a 63-year-old man was reported in Zhejiang, China. Once diagnosed, vancomycin treatment cleared the infections. The patient had no remarkable medical history, and the inspection showed no overt gastrointestinal symptoms, though *C. difficile* was detected in his stool samples. However, we later defined that the *C. difficile* strain isolated from the blood sample was different from the one isolated from his stool using the whole genome sequencing analysis. By retrospective analysis of his medical record, we noticed that the man had a recent tooth extraction thus the bacterium may have invaded through the root canal. Therefore, we suggest that oral *C. difficile* colonization may be a potential risk factor for severe *C. difficile* septicemia, which could be clinically alarming.

## Introduction

*Clostridioides difficile* (also known as *Clostridium difficile*) is a spore-forming anaerobic bacterium that is one of the leading causes of nosocomial and community-acquired infections in many countries (Leffler and Lamont, 2015). *C. difficile* commonly colonizes the colon and causes gastrointestinal diseases and diarrhea. Albeit rare, the bacterium is also capable of causing bloodstream infection resulting in septicemia. *C. difficile* bacteremia first came to notice in 1962 within a 5-month-old infant (Smith and King, 1962). An epidemiological study in a large Canadian health region (population of 1.2 million) between 2000 and 2006 showed that the occurrence of *C. difficile* bacteremia is approximately 0.09 per 100,000 residents per year (Leal et al., 2008). Although detailed pathogenesis remains unknown, *C. difficile* bacteremia normally occurs in patients with known gastrointestinal diseases or other serious underlying diseases and usually are polymicrobial. *C. difficile* polymicrobial sepsis usually happens together with Gram-negative and/or anaerobic bacteremia, most typically gut flora such as *Bacteroides* and *Enterococcus*, suggesting that the bacteremia might be the result of severe gastrointestinal damage (Libby and Bearman, 2009).

Here we report an atypical case of *C. difficile* bacteremia in the patient without notable premorbid symptoms. The whole-genome sequencing analysis showed that it is unlikely a gut-derive infection. By reviewing the medical record, we suggest that *C. difficile* possibly entered the bloodstream through the root canal of the patient in a recent dental surgery. This is an atypical example of the monomicrobial bloodstream infection caused by *C. difficile* in a patient without evidence of concomitant gastrointestinal disease or other severe underlying diseases, which could be clinically alarming.

## Results

**Figure 1:**
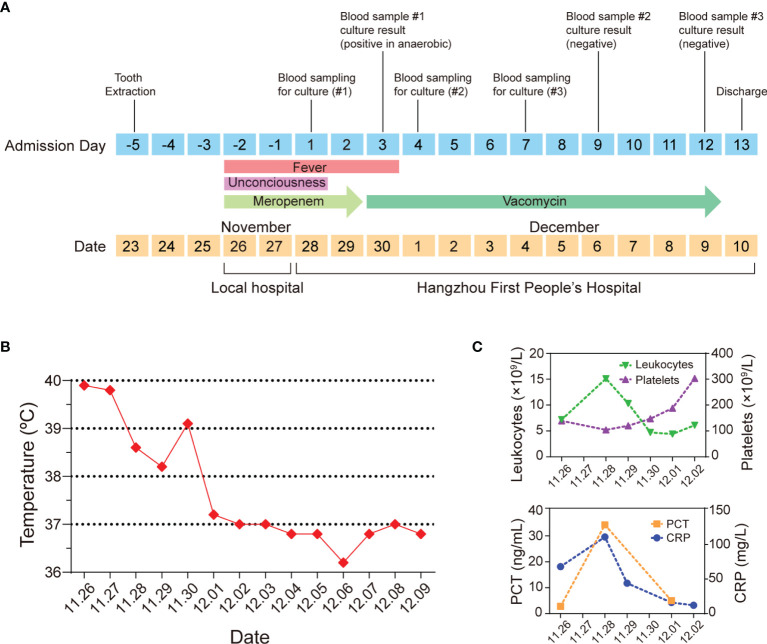
An atypical case of a 63-year-old man with *C. difficile* septicemia in Zhejiang, China. **(A)** Timeline of the main events of the case. **(B)** Clinical evolution of fever. **(C)** Clinical data of leukocytes, platelets, PCT, and CRP.

On November 28, 2020, a 63-year-old man presented to the emergency department of the Affiliated Hangzhou Frist People’s Hospital, Zhejiang University School of Medicine (Hangzhou, China), with a 3-day high fever and 15-hour unconsciousness (**Figure 1A**). The patient reported no vomiting, diarrhea, or abdominal pain, and had no remarkable medical history except for gout. The patient also reported that he had a scalp trauma one month ago and took cefixime for about 10 days to prevent infection in a local hospital. He had a tooth extraction due to dental caries on November 23 in a local clinic. The tooth extraction was not going well initially, thus the operation took as long as 4 h. Two days later, his body temperature elevated to 39.9°C accompanied by restlessness and intermittent delirium. He sought care at a local hospital and received 2 days of meropenem treatment, but his condition deteriorated. He was then transferred to the Hangzhou Frist People’s Hospital, a general hospital in Hangzhou.

On admission, he had sepsis and the sequential organ failure assessment (SOFA) score reached 3 (Singer et al., 2016), his PaO_2_/FiO_2_ was 252 mmHg. The routine blood test showed that his platelet count was 104 × 10^9^/L and total leukocyte count reached 15.1 × 10^9^/L, most of which were neutrophils (92.6%), C-reactive protein (CRP) was up to 111 mg/L, and Procalcitonin (PCT) concentration was 34.32 ng/ml (**Figure 1C**), indicating the patient had an acute bacterial infection. Computed tomography showed no significant abnormalities in the chest, abdomen, and brain. Cardiac ultrasound showed no infective endocarditis. His peripheral blood was sampled and split into four vials for the peripheral-blood culture tests (two anaerobic and two aerobic). Because the patient and his family members were reluctant to have a lumbar puncture, cerebrospinal fluid was not sampled.

Because of the sepsis symptoms such as fever (over 38.6°C), empiric meropenem (1 g administered intravenously every 8 h) was kept being given. On days 1 and 2 of hospitalization, the patient still had a continuous fever (**Figure 1**). On hospital day 3, the blood sample cultured in two anaerobic bottles showed a Gram-positive bacillus signature which was subsequently identified as *C. difficile* using Matrix-Assisted Laser Desorption Ionization-Time of Flight Mass Spectrometry (MALDI-TOF MS). Two aerobic blood cultures showed negative signals. Based on the peripheral-blood culture test result, the patient was diagnosed with monomicrobial *C. difficile* septicemia. In this case, meropenem treatment was immediately stopped and vancomycin was applied (1 g administered intravenously every 12 h) following the clinical instruction for treating *C. difficile* infection (Libby and Bearman, 2009; Kelly et al., 2021).

**Figure 2:**
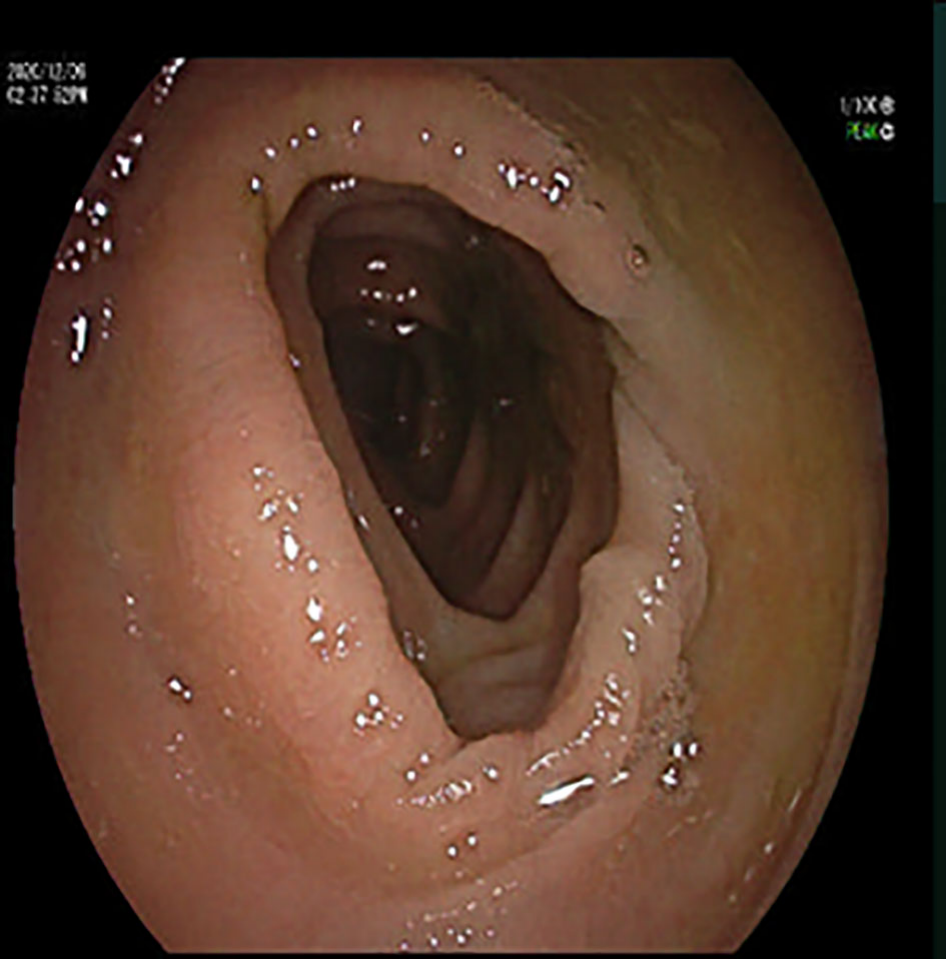
Colonoscopy image: colonoscopy showed a smooth colonic mucosa surface, clear vascular texture, and no erosion or ulcers.

On hospital day 4, the body temperature of the patient turned back to normal, and all clinical parameters were improved. On hospital day 3, a fecal *C. difficile* test showed positive. On hospital day 10, fecal *C. difficile* test results became negative. Colonoscopy was performed on hospital day 12, and no abnormality was observed (**Figure 2**). On hospital day 12, considering that the blood culture test was negative for two consecutive times, the intravenous vancomycin administration was terminated. On hospital day 13, the patient was discharged, and all symptoms had resolved. The patient was satisfied with the treatment outcome and had good compliance with follow-up. The patient had not been hospitalized due to infection since he was discharged.

**Figure 3:**
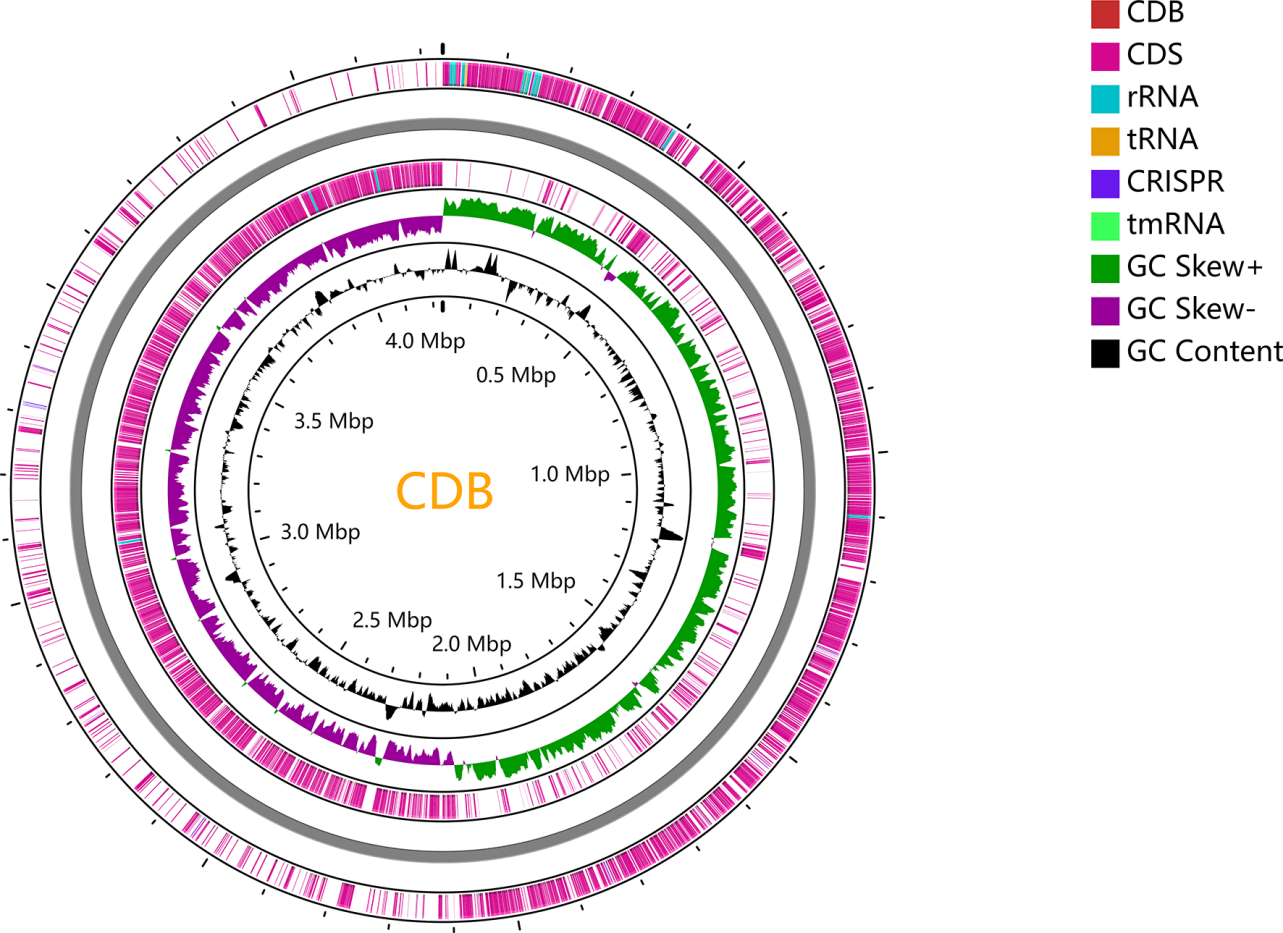
Circular genome map of CDB. The circular genome has been generated with CGView.

**Figure 4:**
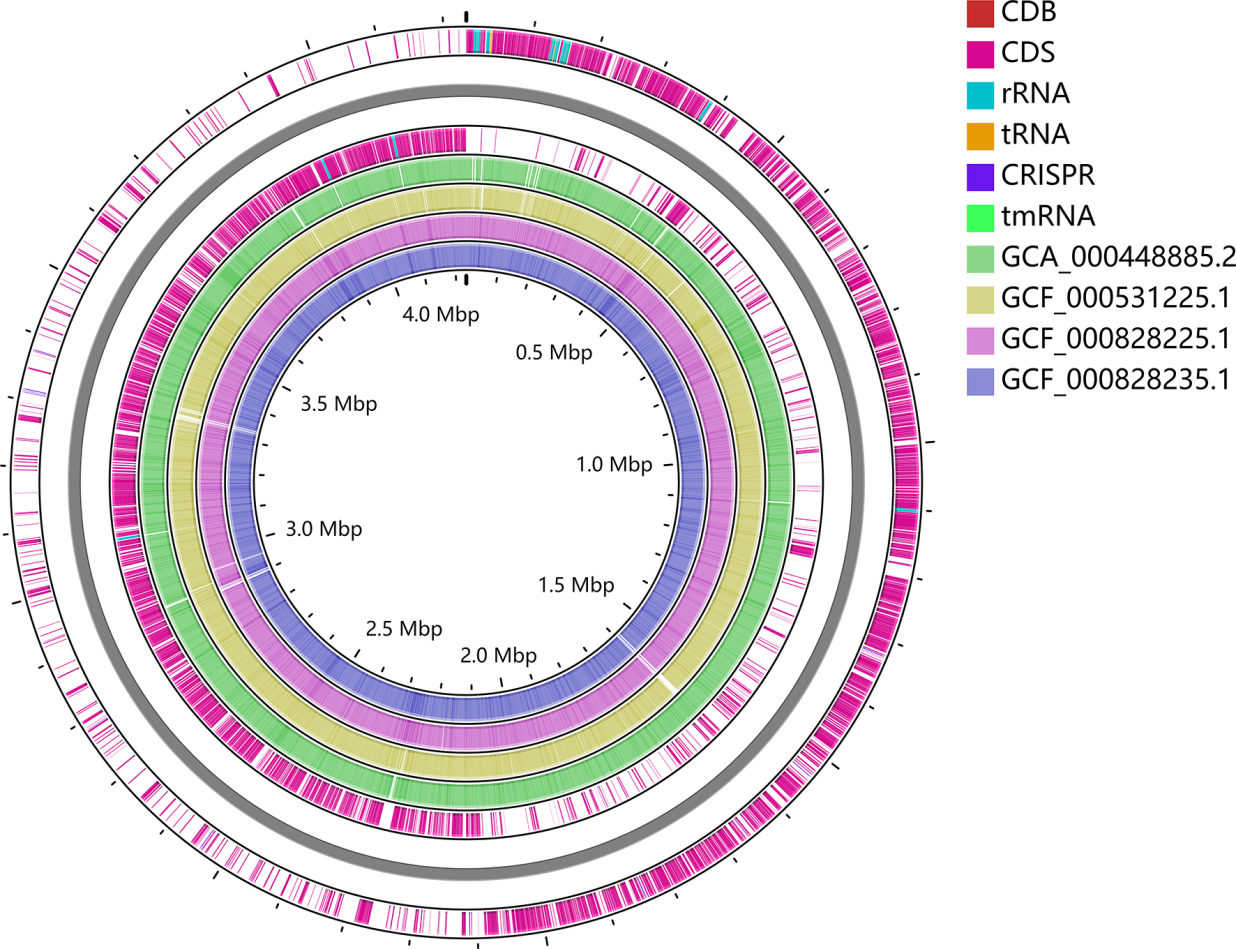
Whole-genome alignment of CDB with four reference *C. difficile* ST54 strains obtained from the public database. The circular genome has been generated using CGView.

To trace the infection route of *C. difficile* in blood, sequence typing analysis of *C. difficile* strains isolated from the blood (CDB) and feces (CDF) was conducted using multilocus sequence typing (MLST) based on the public database (https://pubmlst.org) (Jolley et al., 2018). Interestingly, an MLST analysis showed that CDB belongs to ST54/RT012 (**Figure 3**), while *C. difficile* from the feces belongs to ST3/RT001. ST54/RT012 strains are commonly found in East Asia (Liao et al., 2018; Shen et al., 2020; Knight et al., 2021). We performed genome alignment among the CDB and other four references ST54/RT012 strains but no major genomic divergence was defined (**Figure 4**).

Drug susceptibility tests showed that CDB is susceptible to chloramphenicol, piperacillin, metronidazole, moxifloxacin, meropenem, vancomycin, and intermediate to ampicillin but resistant to clindamycin (**Supplementary Table 1**). The interpretation was based on the minimal inhibitory concentration (MIC) breakpoints for anaerobic bacteria or *C. difficile* (CLSI, 2021; EUCAST, 2021). Particularly, meropenem was used in the initial treatment but failed to cure the patient, how CDB tolerated meropenem *in vivo* is a critical question that remains to be further studied.

## Discussion

Here we reported an atypical case of monomicrobial *C. difficile* septicemia in a 63-year-old premorbid healthy man without gastrointestinal symptoms. Since CBD and CDF belong to different STs and no overt pathological phenomenon was found in colonoscopy, CDB may not be derived from the gut. Contrast-enhanced computed tomography of the chest and abdomen was done, but no cyst was found. A colonoscopy was applied, and the result was normal. Cardiac ultrasound showed no infective endocarditis. Previously, Henriques et al. showed that *C. difficile* was the fourth mean count of the 107 test species in amplified root canal samples taken from 40 teeth (Henriques et al., 2016). As this patient experienced a four-hour tooth extraction three days before getting the fever, we suspected that *C. difficile* may have invaded his body through the root canal during the oral operation. Unfortunately, the tooth extraction was performed days ago, and the extracted tooth had already been discarded. In addition, sampling from the root canal is traumatic and the patient had already received Vancomycin treatment. Thus, we were unable to experimentally validate our hypothesis.

Notably, the patient had a scalp abrasion one month before and had been taking cefixime for 10 days. This could be another possible factor of developing *C. difficile* sepsis for the patient. It was shown that the sepsis risk was 65% higher in the population pre-exposed to high-risk antibiotics (3rd and 4th generation cephalosporins, carbapenems, and fluoroquinolones) than those who did not receive antibiotics (Baggs et al., 2018).

In recent years, there is rising attention to the association between *C. difficile* infection (CDI) and sepsis. The risk of severe sepsis after hospitalization with CDI was 70% greater than an infection-associated hospitalization without CDI (Prescott et al., 2015). Unlike canonical *C. difficile* infection, bacteremia could lead to severe illness and high death rates. A previous study reported that 42% of the patients with *C. difficile* bacteremia died in Taiwan, China (Lee et al., 2010). Falcone et al. suggested that ribotype 027 infection, CDI recurrence, severe CDI, and oral vancomycin (at >500 mg/day) were independent risk factors associated with the development of nosocomial bloodstream infections after CDI. Among the 393 cases of CDI analyzed in their study, 72 developed a primary nosocomial BSI (bloodstream infection), while 321 had CDI without microbiological and clinical evidence of BSI (Falcone et al., 2016).

*C. difficile* normally colonizes the gastrointestinal tract and causes diseases. Extraintestinal CDI, which is mostly associated with substantial illness, comprises only 0.17% of all CDI cases (Mattila et al., 2013). Chung et al. reported that extraintestinal CDI was uncommonly associated with *C. difficile*-induced colitis (Chung et al., 2020). The gastrointestinal disruption caused by malignancy and aging was associated with *C. difficile* bacteremia, and extraintestinal CDI often occurs in patients with surgical manipulation of the gastrointestinal tract (Gupta et al., 2014). Gut microbiome disruption with related CDI may predispose patients to sepsis, which could be an important area to be studied (Adelman et al., 2020).

In this case, the patient had *C. difficile* inhabited in his gut yet was asymptomatic, and the *C. difficile* strains isolated in the blood and feces were different. The patient might have more than one *C. difficile* strain in his feces but the possibility is low, as multiple colonies were validated. Therefore, the CDB is unlikely derived from the gut but somewhere else such as the deep layer of the oral microbiome. In closing, we suggest that oral operations with the deep wound may also cause *C. difficile* septicemia thus need to be alerted.

## Methods

### Isolation of *C. difficile* From the Feces

To isolate *C. difficile* from the feces, 200 μl of diarrheal and 800 μl of ethanol were mixed and homogenized by vortexing. After the incubation for 1 h, the mixture was centrifuged at 8,000 rpm for 4 min and the supernatant was discarded. The pellet was resuspended in 500 μl of stroke-physiological saline solution and then spread onto the cycloserine-cefoxitin fructose agar plates (Oxoid, Basingstoke, UK). The plates were placed in the anaerobic tank (Anoxomat Mark II) and allowed to grow at 37°C. After 48 h, 5 colonies were randomly selected and applied to the following MALDI-TOF MS analysis and genome-sequencing.

### Whole-Genome Sequencing and Sequence Analysis

The *C. difficile* genome was extracted using a TIANamp Bacteria DNA Kit. The whole-genome sequencing and assembling were accomplished by using Pacbio supplemented with Illumina MiniSeq. The whole-genome sequence was submitted to PubMLST (https://pubmlst.org) for sequence typing. The genome alignment of CDB with four other ST54 strains and the figure of the whole genome sequence of CDB was generated using CGView Server (http://cgview.ca/) (Grant and Stothard, 2008). The genome sequence of CDB has been deposited to the NCBI database Bio-project PRJNA790702.

### Drug Susceptibility Test

Chloramphenicol, ampicillin, metronidazole, moxifloxacin, ciprofloxacin, levofloxacin, vancomycin, piperacillin, clindamycin, erythromycin, and kanamycin were selected to test their MIC against CDB. The seeding CDB culture was grown in BHI medium in the anaerobic chamber overnight, and the culture was added to 96-well plates loaded with gradient concentrations of corresponding antibiotics with a seed volume of 1%. The growth of CDB in the 96-well plates was monitored daily, the final MIC results were based on the growth of CDB after 2 days of culturing.

## Data Availability Statement

The datasets presented in this study can be found in online repositories. The name of the repository and accession number can be found below: NCBI; PRJNA790702.

## Ethics Statement

The studies involving human participants were reviewed and approved by the Ethics Committee of Affiliated Hangzhou First People’s Hospital, Zhejiang University School of Medicine. The patients/participants provided their written informed consent to participate in this study.

## Author Contributions

LW and LT initiated and designed the project LW and ZC collected the clinical data LW, DL, and LH performed the experiment and data analysis LW, DL, and LT wrote the manuscript XW and LT revised the manuscript for important intellectual content All authors listed have made a substantial, direct, and intellectual contribution to the work and approved it for publication.

## Funding

This study was partially supported by the National Natural Science Foundation of China (Grant no. 31970129 to LT). LT also acknowledges support from the Zhejiang Provincial Natural Science Foundation of China under Grant no. LR20C010001, the Westlake Education Foundation, and the Westlake Laboratory of Life Sciences and Biomedicine. LW acknowledges support from the Zhejiang Provincial Medical and Health Technology Project (Grant no. 2021RC105).

## Conflict of Interest

The authors declare that the research was conducted in the absence of any commercial or financial relationships that could be construed as a potential conflict of interest.

## Publisher’s Note

All claims expressed in this article are solely those of the authors and do not necessarily represent those of their affiliated organizations, or those of the publisher, the editors and the reviewers. Any product that may be evaluated in this article, or claim that may be made by its manufacturer, is not guaranteed or endorsed by the publisher.

## Supplementary Material

Supplementary Table 1 Drug susceptibility testing results of CDB. *R, resistant; S, susceptible; I, intermediate.
