# Supplementary material for: An Atypical Case of Monomicrobial Clostridioides difficile Septicemia With No Gastrointestinal Manifestations
Source: Front Cell Infect Microbiol. 2022 Mar 31;12:853252. doi: 10.3389/fcimb.2022.853252 (PMC9009443; doi:10.3389/fcimb.2022.853252)
Supplement: Supplementary Table 1 — Drug susceptibility testing results of CDB. *R, resistant; S, susceptible; I, intermediate. [file Table_1.pdf]

Supplementary Table 1. Drug susceptibility testing results of CDB.

**Antimicrobial drug**	**MIC (μg/mL)**	**Interpretation ***
Chloramphenicol	5	S
Ampicillin	1	I
Piperacillin	15	S
Metronidazole	0.75	S
Moxifloxacin	1.25	S
Clindamycin	>32	R
Meropenem	1	S
Vancomycin	1	S

*R, resistant; S, susceptible; I, intermediate.
